# PSYCHOLOGICAL RESILIENCE MAY IMPROVE SLEEP QUALITY OF FAMILY CAREGIVERS OF OLDER ADULTS

**DOI:** 10.1093/geroni/igae098.0253

**Published:** 2024-12-31

**Authors:** Rahul Malhotra, Ha-Linh Quach, Jeremy Lim-Soh

**Affiliations:** Duke-NUS Medical School, Singapore, Singapore; Duke-NUS CARE, Singapore, Singapore; Duke-NUS Medical School, Singapore, Singapore

## Abstract

Given the increasing focus on the importance of sleep for health and well-being, we assessed the (a) association of caregiving status (family caregiver/non-caregiver) with sleep quality and its moderation by psychological resilience and (b) factors influencing sleep quality among caregivers. Cross-sectional data on subjective sleep quality (Pittsburgh Sleep Quality Index (PSQI); higher global or component scores represent worse sleep quality) and psychological resilience (Connor-Davidson resilience scale) from Singapore on 167 family caregivers of older adults and 135 non-caregivers was analyzed. Multivariable linear regression models assessed the association of caregiving status with global PSQI score and it’s moderation by psychological resilience and identified caregiver and care-recipient characteristics associated with global PSQI score among caregivers. Mean global PSQI score (6.0 versus 4.5), proportion with poor sleep quality (global PSQI score>5; 46.1% versus 28.1%) and mean component scores (for sleep latency, sleep quality, sleep duration, and daytime dysfunction) were higher for caregivers, versus non-caregivers, reflecting their worse sleep quality. In multivariable analysis, caregivers had higher global PSQI score than non-caregivers (beta-coefficient: 1.04, p<0.05). However, this association was moderated by psychological resilience (beta-coefficient: -0.70, p<0.05) – the deficit in sleep quality for caregivers versus non-caregivers reduced with higher psychological resilience. Finally, among caregivers, those with higher education, fewer chronic conditions, fewer functional limitations, and higher psychological resilience had lower global PSQI score, i.e., better sleep quality. Our findings reveal the detrimental effect of being a family caregiver (versus not) on sleep quality and identify psychological resilience as a potential modifiable factor for mitigating it.

